# Supplementary material for: Closed State Structure of the Pore Revealed by Uncoupled Shaker K+ Channel
Source: bioRxiv. 2025 Mar 17:2025.03.17.643777. Preprint. [Version 1] doi: 10.1101/2025.03.17.643777 (PMC11956924; doi:10.1101/2025.03.17.643777)
Supplement: 1 [file NIHPP2025.03.17.643777V1-supplement-1.pdf]

bioRxiv preprint doi: https://doi.org/10.1101/2025.03.17.643777; this version posted March 17, 2025. The copyright holder for this preprint (which was not certified by peer review) is the author/funder, who has granted bioRxiv a license to display the preprint in perpetuity. It is made available under aCC-BY 4.0 International license.

Supplementary Figure 1

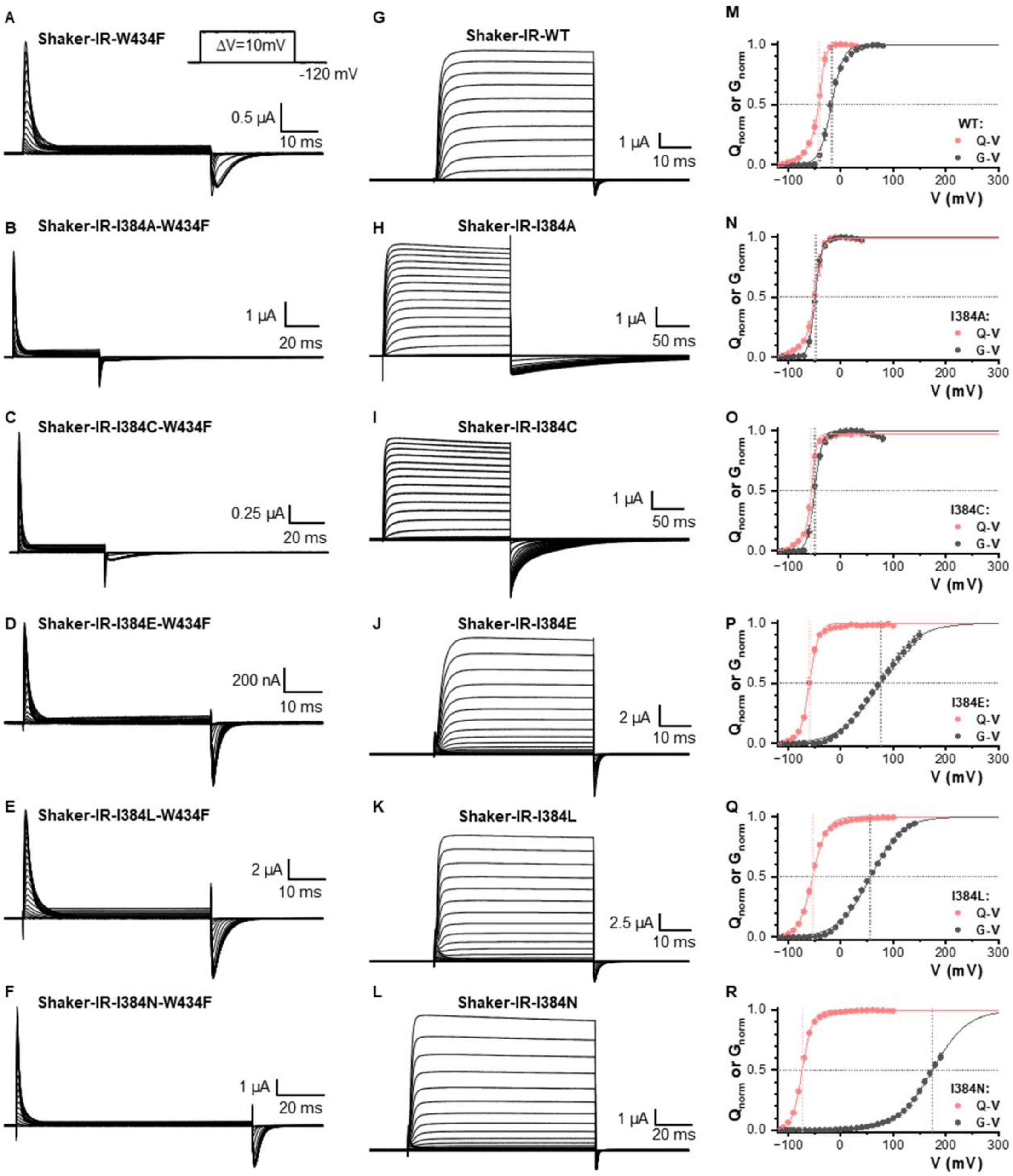

Supplementary Figure 1. Gating current, ionic current and QV-GV curve comparison for all the I384 mutants in Shaker-IR potassium channel. Representative gating currents for WT **(A)**, I384A **(B)**, I384C **(C)**, I384E **(D)**, I384L **(E)**, and I384N **(F)**. Representative ionic currents for WT **(G)**, I384A **(H)**, I384C **(I)**, I384E **(J)**, I384L **(K)**, and I384N **(L)**. Note that the gating current experiments were conducted with W434F background. The comparison of the QV/GV WT **(M)** curve indicates that I384A **(N)** and I384C **(O)** strengthen the coupling while I384E **(P)**, I384L **(Q)** and I384N **(R)** weaken the coupling.

bioRxiv preprint doi: https://doi.org/10.1101/2025.03.17.643777; this version posted March 17, 2025. The copyright holder for this preprint (which was not certified by peer review) is the author/funder, who has granted bioRxiv a license to display the preprint in perpetuity. It is made available under aCC-BY 4.0 International license.

Supplementary Figure 2

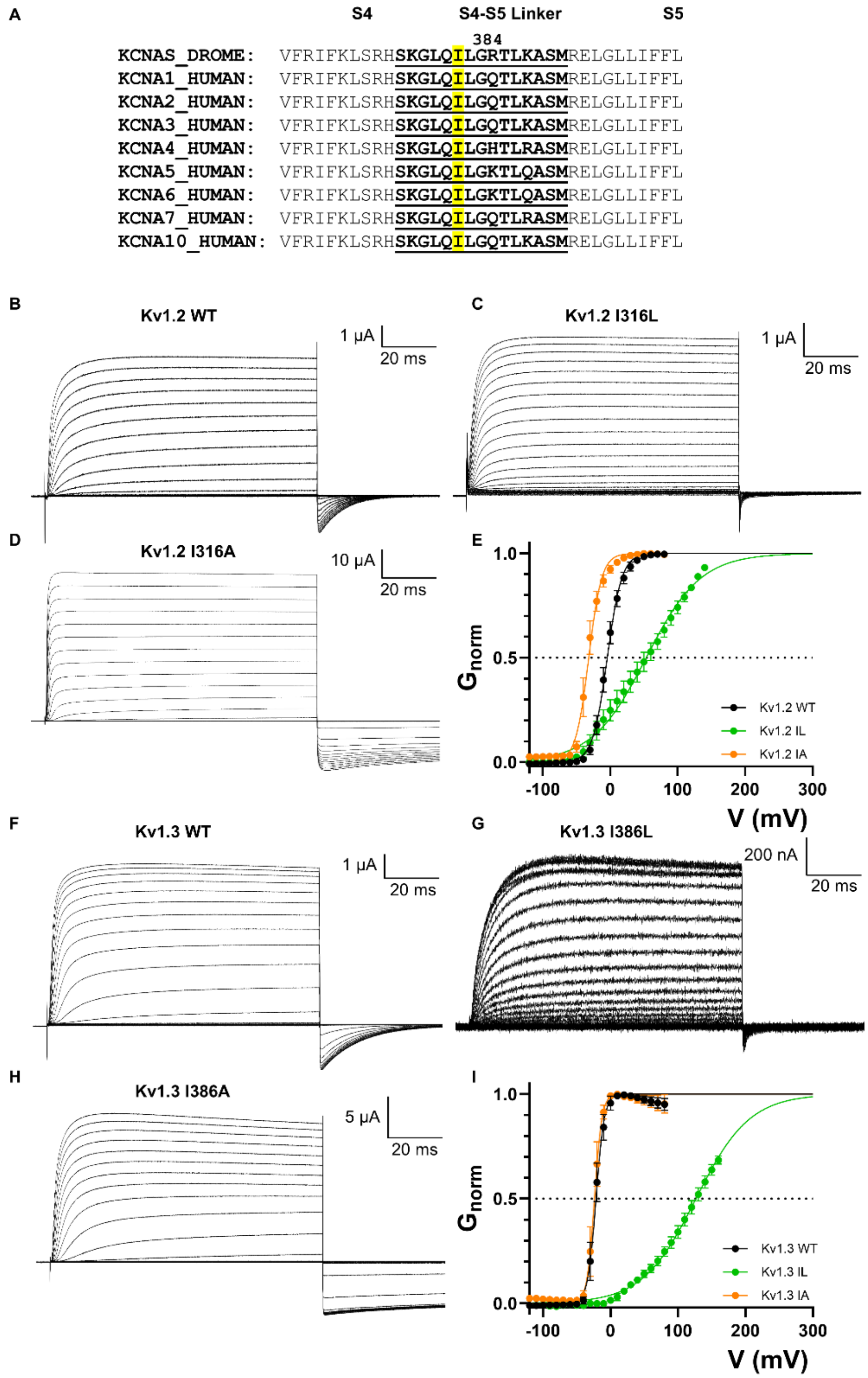

Supplementary Figure 2. Conserved Isoleucine that controls EMC in Kv1 families. **A)** Sequence alignment of the Shaker potassium channel with human Kv1 channels. I384 is conserved across all the channels. **B)-D)** Results from hKv1.2 mutating the equivalent isoleucine. **F)-I)** Results from hKv1.3 mutating the equivalent isoleucine. Similar behaviors were seen in both channels.

bioRxiv preprint doi: https://doi.org/10.1101/2025.03.17.643777; this version posted March 17, 2025. The copyright holder for this preprint (which was not certified by peer review) is the author/funder, who has granted bioRxiv a license to display the preprint in perpetuity. It is made available under aCC-BY 4.0 International license.

Supplementary Figure 3

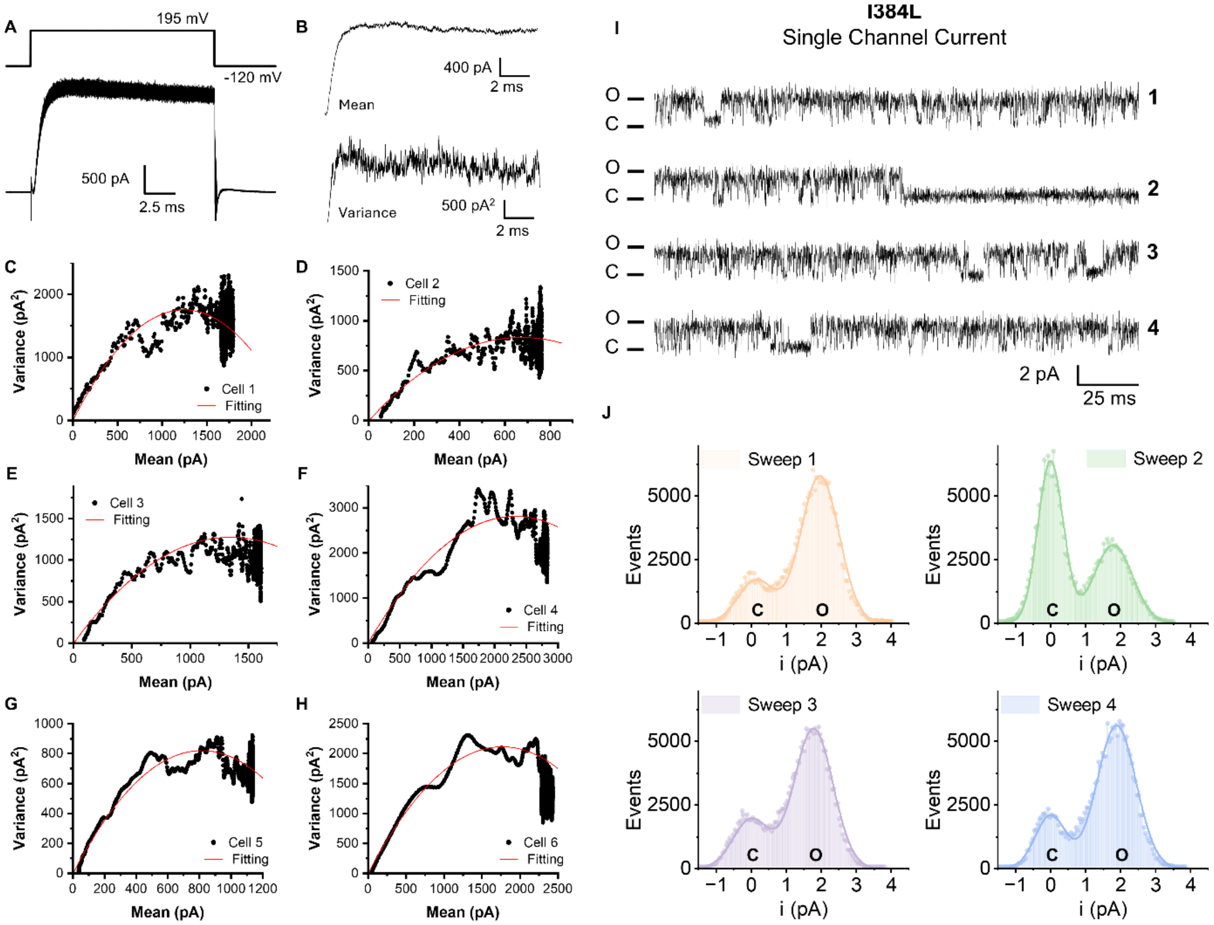

Supplementary Figure 3. Noise analysis and Single channel recording of I384L. Six independent noise analysis experiments were shown with their fitting parameters **(A-H)**. Voltage protocol is inset in **A**. Representative 150 current traces overlapped are shown in **A)** and in **B)** is depicted the Mean and variance from those recordings. **I)** Four representative current traces for single channel records at 195mV, and increased flickering behavior was observed. **J)** Histogram of the single channel recording traces shown in **I)**.

bioRxiv preprint doi: https://doi.org/10.1101/2025.03.17.643777; this version posted March 17, 2025. The copyright holder for this preprint (which was not certified by peer review) is the author/funder, who has granted bioRxiv a license to display the preprint in perpetuity. It is made available under aCC-BY 4.0 International license.

Supplementary Figure 4

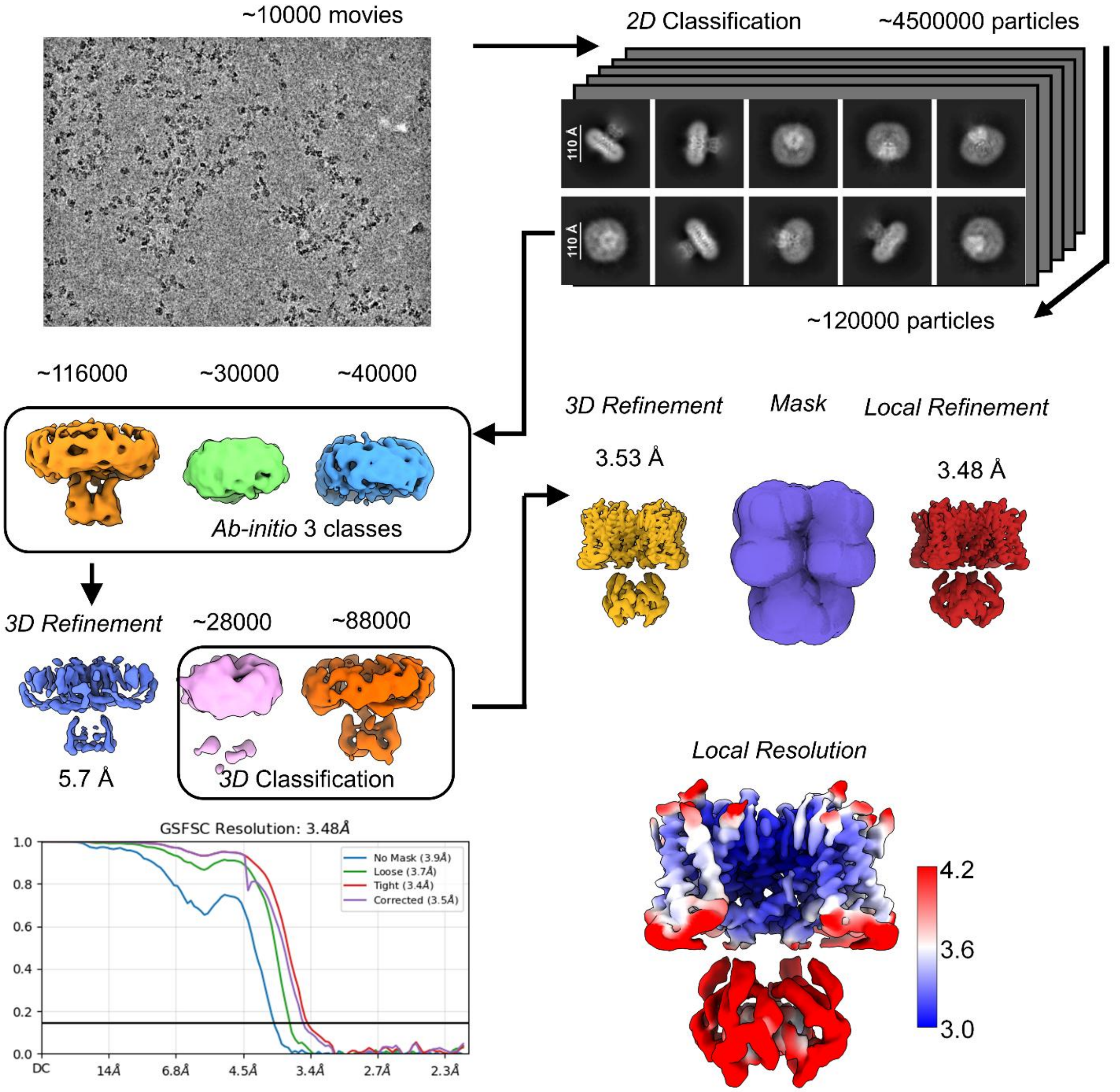

Supplementary Figure 4. Pipeline of the structural determination for Shaker-IR-I384R. Resolution curve and local resolution map.

bioRxiv preprint doi: https://doi.org/10.1101/2025.03.17.643777; this version posted March 17, 2025. The copyright holder for this preprint (which was not certified by peer review) is the author/funder, who has granted bioRxiv a license to display the preprint in perpetuity. It is made available under aCC-BY 4.0 International license.

Supplementary Figure 5

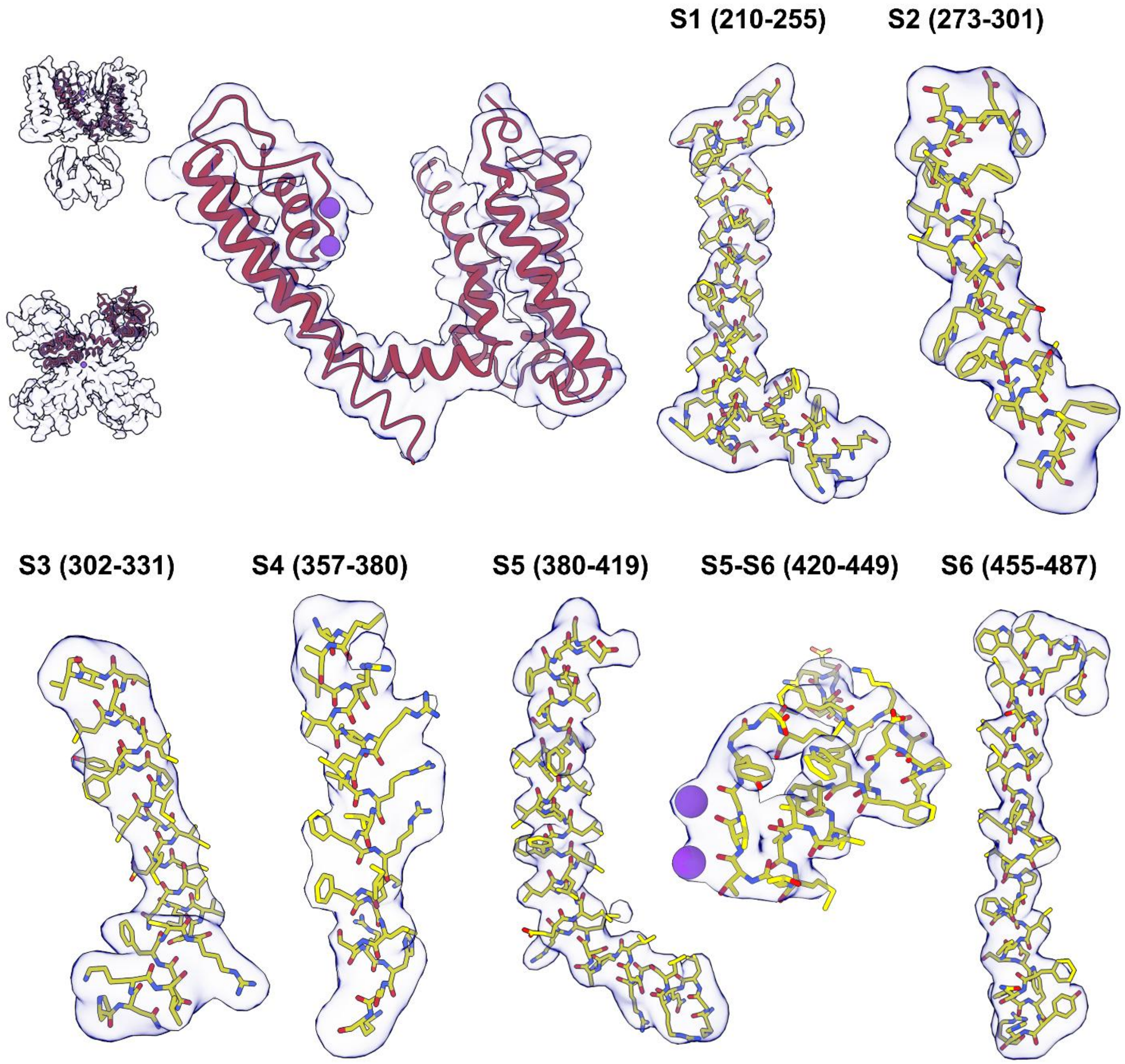

Supplementary Figure 5. Comparison of atomic model and the density.

bioRxiv preprint doi: https://doi.org/10.1101/2025.03.17.643777; this version posted March 17, 2025. The copyright holder for this preprint (which was not certified by peer review) is the author/funder, who has granted bioRxiv a license to display the preprint in perpetuity. It is made available under aCC-BY 4.0 International license.

Supplementary Figure 6

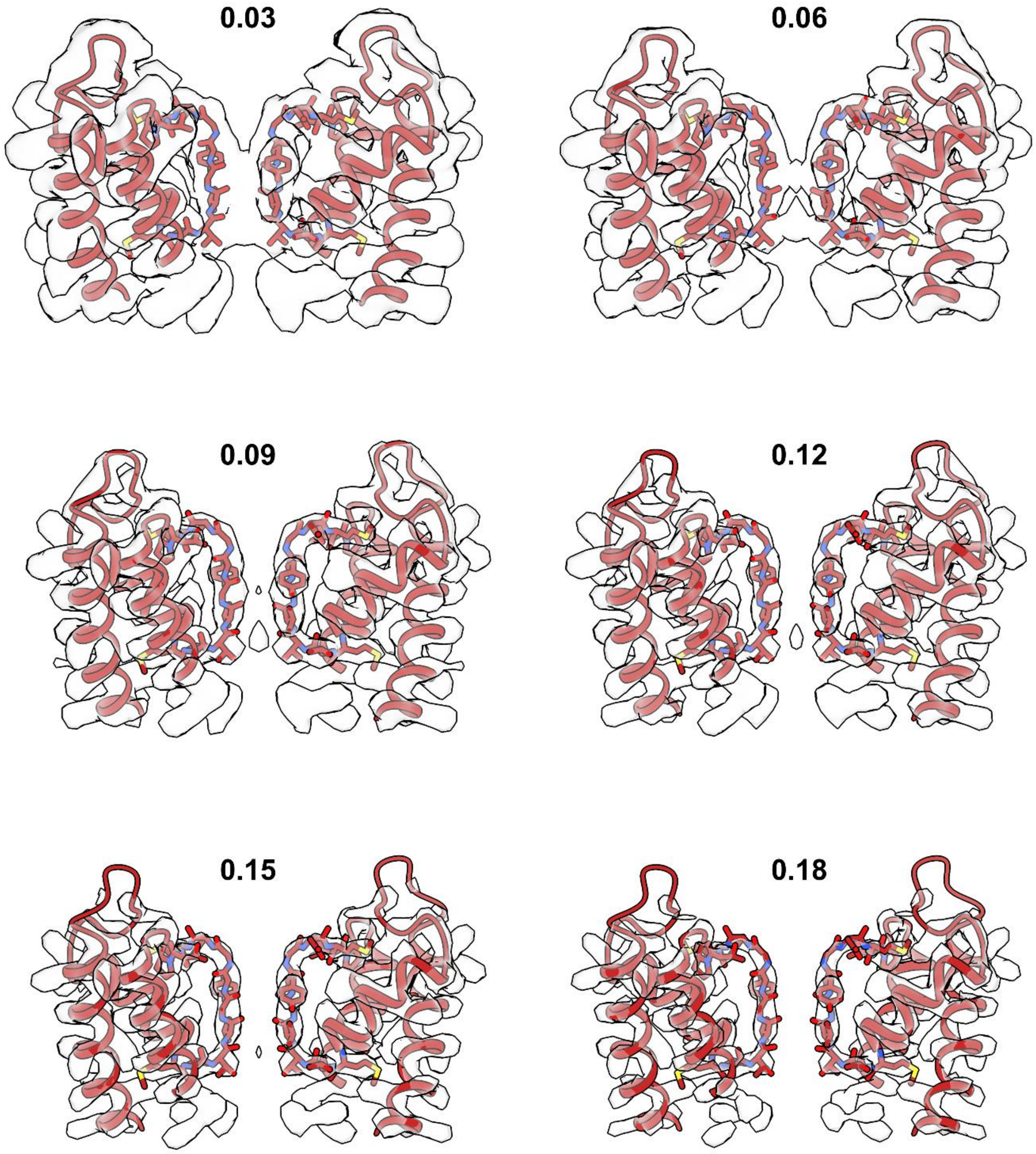

Supplementary Figure 6. Atomic model overlaid with the density map plotted with variable iso-surface value

bioRxiv preprint doi: https://doi.org/10.1101/2025.03.17.643777; this version posted March 17, 2025. The copyright holder for this preprint (which was not certified by peer review) is the author/funder, who has granted bioRxiv a license to display the preprint in perpetuity. It is made available under aCC-BY 4.0 International license.

Supplementary Figure 7

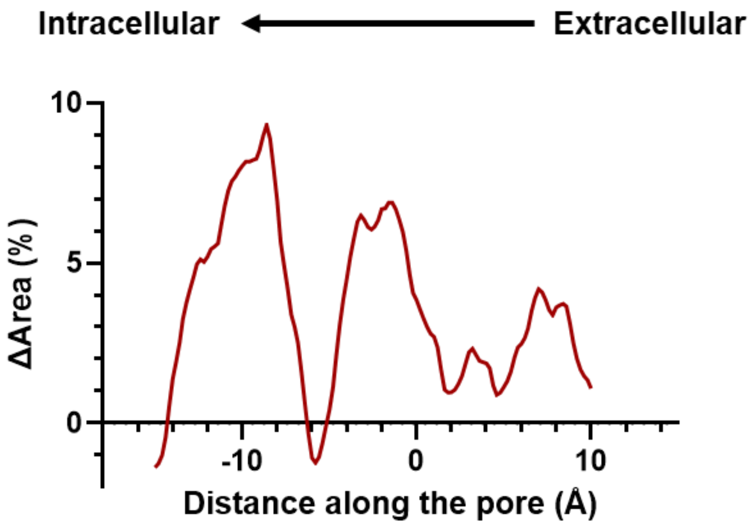

Supplementary Figure 7. Percent area increase the open state compared to the closed state along the membrane. Positive value indicates expansion in open state.

bioRxiv preprint doi: https://doi.org/10.1101/2025.03.17.643777; this version posted March 17, 2025. The copyright holder for this preprint (which was not certified by peer review) is the author/funder, who has granted bioRxiv a license to display the preprint in perpetuity. It is made available under aCC-BY 4.0 International license.

Supplementary Figure 8

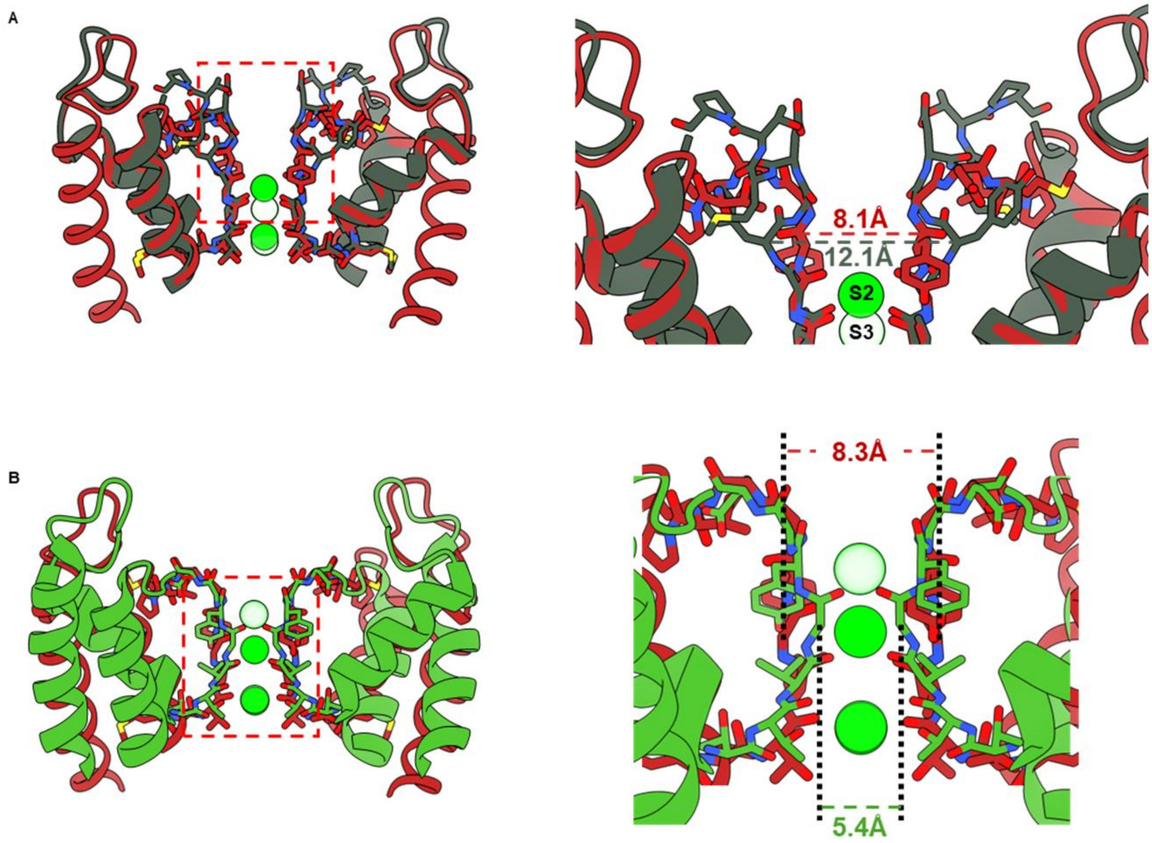

Supplementary Figure 8. Comparison of the noncanonical filter with dilated filter, **A), B)** (PDB:8ETO) and pinched filter, **C), D)** (PDB:3F5W). The noncanonical filter is different from both conformations.

bioRxiv preprint doi: https://doi.org/10.1101/2025.03.17.643777; this version posted March 17, 2025. The copyright holder for this preprint (which was not certified by peer review) is the author/funder, who has granted bioRxiv a license to display the preprint in perpetuity. It is made available under aCC-BY 4.0 International license.

Supplementary Figure 9

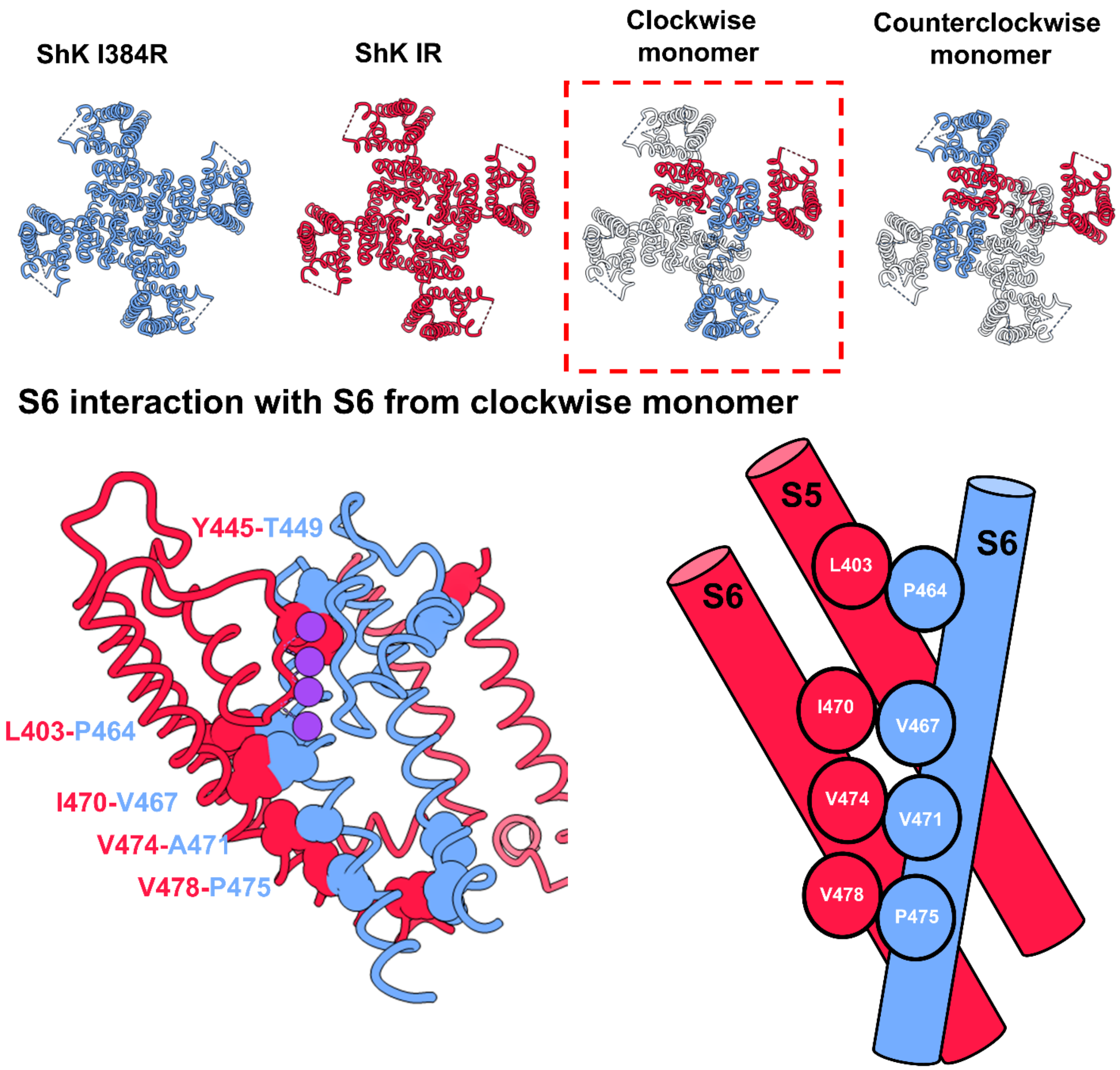

Supplementary Figure 9. Clash analysis by assembling open state and closed state structure into the same homotetramer. The lower panel shows the pair clashes happened between the clockwise monomer of open and closed state. Those pairs of residues likely are involved in the activation of the pore.

bioRxiv preprint doi: https://doi.org/10.1101/2025.03.17.643777; this version posted March 17, 2025. The copyright holder for this preprint (which was not certified by peer review) is the author/funder, who has granted bioRxiv a license to display the preprint in perpetuity. It is made available under aCC-BY 4.0 International license.

Supplementary Figure 10

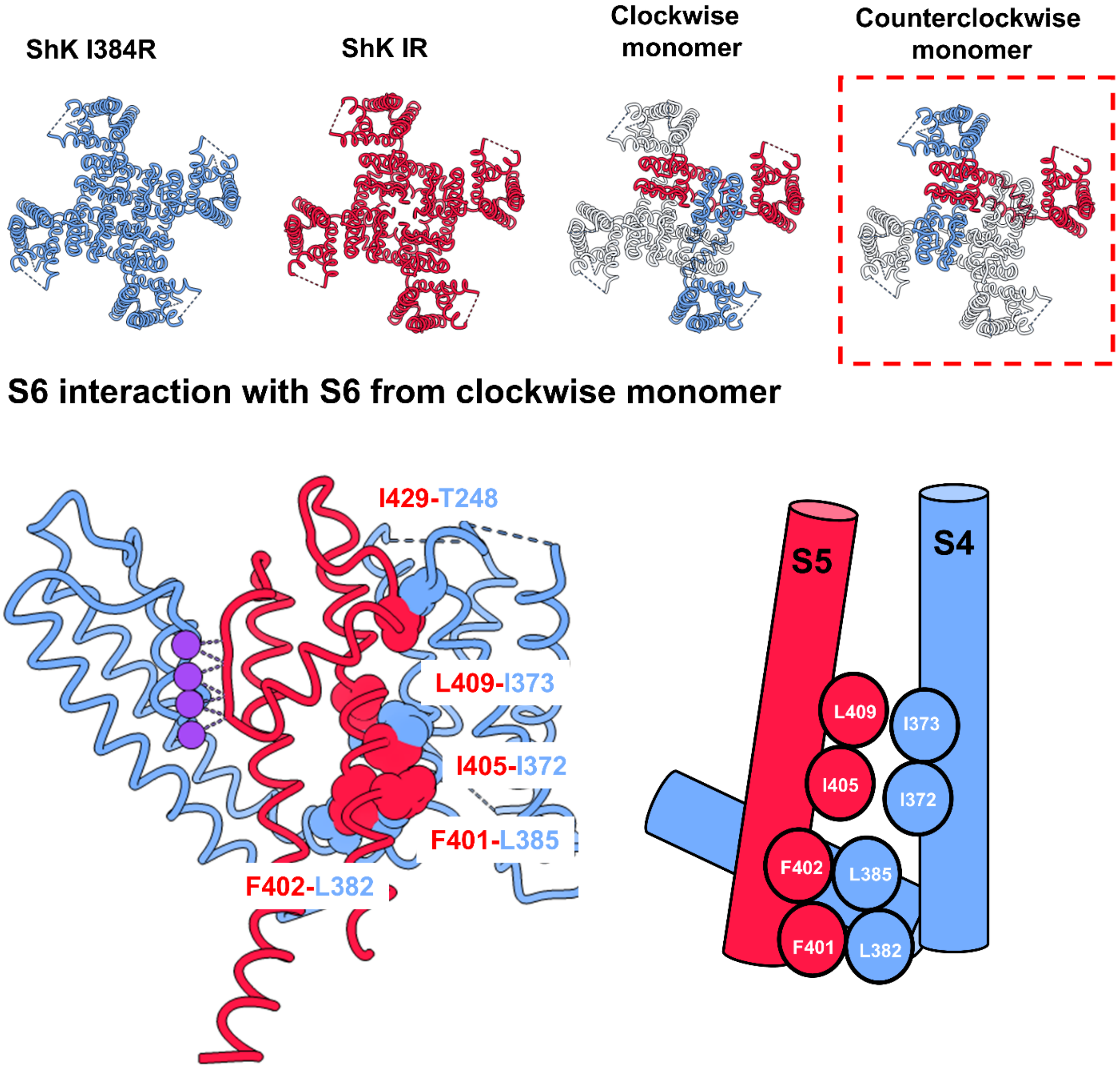

Supplementary Figure 10. Clash analysis by assembling open state and closed state structure into the same homotetramer. The lower panel shows the pair clashes happened between the counterclockwise monomer of open and closed state. Those pairs of residues likely are involved in the electromechanical coupling or the noncanonical coupling.

bioRxiv preprint doi: https://doi.org/10.1101/2025.03.17.643777; this version posted March 17, 2025. The copyright holder for this preprint (which was not certified by peer review) is the author/funder, who has granted bioRxiv a license to display the preprint in perpetuity. It is made available under aCC-BY 4.0 International license.

Supplementary Tables

Table 1: Fitting parameters for GV curves

	$V_{1/2}$			*z*		
WT	-17.70	±	0.73	2.22	±	0.13
I384A	-48.70	±	0.26	4.01	±	0.15
I384C	-49.80	±	0.62	3.64	±	0.29
I384E	74.45	±	0.91	0.70	±	0.02
I384L	56.12	±	0.70	0.84	±	0.02
I384N	174.33	±	0.25	0.76	±	0.01

bioRxiv preprint doi: https://doi.org/10.1101/2025.03.17.643777; this version posted March 17, 2025. The copyright holder for this preprint (which was not certified by peer review) is the author/funder, who has granted bioRxiv a license to display the preprint in perpetuity. It is made available under aCC-BY 4.0 International license.

Table 2: Fitting parameters for QV curves

	V1			***z1***			***v2***			***z2***			***N***		
WT	-62.20	±	1.08	2.10	±	0.14	-39.26	±	0.41	4.51	±	0.22	0.15	±	0.01
I384A	-63.81	±	3.84	1.46	±	0.29	-49.09	±	1.79	3.39	±	0.34	0.20	±	0.02
I384C	-69.31	±	3.07	1.79	±	0.30	-56.26	±	1.26	5.19	±	0.80	0.14	±	0.02

	$V_{1/2}$			***z***		
I384E	-59.92	±	0.51	2.84	±	0.14
I384L	-53.89	±	0.56	1.92	±	0.07
I384N	-73.32	±	0.33	2.61	±	0.08
I384R	-76.82	±	0.24	2.85	±	0.07

bioRxiv preprint doi: https://doi.org/10.1101/2025.03.17.643777; this version posted March 17, 2025. The copyright holder for this preprint (which was not certified by peer review) is the author/funder, who has granted bioRxiv a license to display the preprint in perpetuity. It is made available under aCC-BY 4.0 International license.

Table 3: Fitting parameters for noise analysis

Cell	***Fitting***			***Vm (mV)***	***Vrev (mV)***	***PoMax***	***Mean (pA)***	***Y (Single)***	***pS***
		**Value**	**Error**						
I384L_04-04-24-001	I (pA)	2.01	0.03	160.00	-57.00	0.70	1139.90	0.00	13.27
	N	809.71	24.69						
I384L_04-04-24-002	I (pA)	2.40	0.03	160.00	-57.00	0.69	2434.20	0.00	16.02
	N	1475.23	37.67						
I384L_04-04-24-003	I (pA)	2.42	0.06	180.00	-57.00	0.61	2835.10	0.00	16.75
	N	1922.18	98.98						
I384L_04-04-24-005	I (pA)	1.89	0.07	180.00	-57.00	0.60	1609.00	0.00	13.38
	N	1421.08	223.78						
I384L_04-04-24-006	I (pA)	2.48	0.07	195.00	-57.00	0.57	764.04	0.00	17.30
	N	543.38	39.13						
I384L_04-04-24-009	I (pA)	2.75	0.04	195.00	-57.00	0.64	500.06	0.00	17.12
	N	285.07	7.87						
I384L_04-12-24-008	I (pA)	2.82	0.07	195.00	-57.00	0.72	1798.00	0.00	15.50
	N	885.83	43.38						

bioRxiv preprint doi: https://doi.org/10.1101/2025.03.17.643777; this version posted March 17, 2025. The copyright holder for this preprint (which was not certified by peer review) is the author/funder, who has granted bioRxiv a license to display the preprint in perpetuity. It is made available under aCC-BY 4.0 International license.

Table 4: Fitting parameters for single channel analysis

	Sweep 1		***Sweep 2***		***Sweep 3***		***Sweep 4***	
	**Value**	**error**	**Value**	**error**	**Value**	**error**	**Value**	**error**
μ1	3.93	0.01	3.75	0.01	3.94	0.01	3.99	0.01
σ1	0.77	0.01	0.78	0.01	0.78	0.01	0.79	0.01
A1	22399.67	243.33	11969.20	182.66	21546.90	235.23	22217.59	255.66
μ2	2.11	0.02	1.97	0.00	2.16	0.02	2.11	0.02
σ2	0.73	0.03	0.51	0.01	0.73	0.03	0.62	0.03
A2	6187.80	238.43	16331.76	150.59	7126.41	229.39	6420.97	228.52
